# Ocular toxicity of Calotropis - missing links

**DOI:** 10.4103/0301-4738.60074

**Published:** 2010

**Authors:** Sujata Lakhtakia, P C Dwivedi, Pankaj Choudhary, Charudatt Chalisgaonkar, Jainendra Rahud

**Affiliations:** Department of Ophthalmology, SS Medical College, Rewa - 486 001, MP, India

Dear Editor,

We read with great interest the article titled “Ocular toxicity by latex of *Calotropis procera* (Sodom apple)” by Basak *et al*.[1]

Use of Calotropis for worshipping Lord Shiva is fairly common in our region (Eastern Madhya Pradesh) and also in the adjoining areas of Uttar Pradesh. As a result, we too get to see many cases of Calotropis-induced ocular inflammation and this had prompted us to conduct a study on the same (presented as Poster no. 049 entitled “Spectrum Of Ocular Manifestations Of Calotropis Induced Chemical Injury” in the 67^th^ All India Ophthalmology Conference, 5-8 February, 2009, Jaipur).

We studied 47 patients reporting to the Ophthalmology Department between June 2005 and May 2008, all with a positive history of contact with Calotropis latex. In our study, females were more affected (70%) as against male preponderance seen in the study of Basak *et al*.[1] A probable explanation for this could be that females are more involved in worshipping rites. Slit lamp examination showed dermatitis in 63%, conjunctivitis in 55%, keratitis with Descemet's folds in 36% and keratouveitis in 9% of the cases. Secondary glaucoma was not seen in any patient.

All patients were treated with topical antibiotics, steroids, cycloplegics and lubricants. Most patients showed a dramatic response in terms of symptomatic comfort and best-corrected visual acuity.

During the course of our study, we performed an exhaustive search of the published literature for related studies. Besides foreign case reports, we also came across three similar studies/case reports from India[2–4] and one from Saudi Arabia,[5] which are mentioned in the references below.

Unfortunately, there is no mention of these case reports/studies in the article by Basak *et al*.[1]

To conclude, Calotropis-induced ocular inflammation is not of infrequent occurrence in the Indian scenario and may be associated with keratouveitis. Thus, it becomes imperative for ophthalmologists to entertain a high index of suspicion for Calotropis toxicity and elicit a relevant history of contact in patients with such clinical presentation.
